# Gender differences: it’s time for a rational approach

**DOI:** 10.1007/s12471-019-1277-7

**Published:** 2019-04-09

**Authors:** J. J. Piek

**Affiliations:** Department of Cardiology, Academic Medical Center, Amsterdam, The Netherlands

The present issue of the Netherlands Heart Journal focusses on gender differences in coronary artery disease. The development of coronary artery disease is markedly different between men and women [1]. In general, in men, with risk factors, coronary artery disease develops gradually over decades leading to symptoms, typically, at the age of 60. In women, the onset of coronary atherosclerosis starts later and accelerates after menopause. Rare complications such as spontaneous coronary dissections and Takotsubo syndrome occur much more frequently in women, as outlined in this issue [2]. However, vasospastic angina, either caused by epicardial or microvascular vasospasm, is a more frequent phenomenon that affects women more often than men [3].

Recent studies from the group of Ong et al. show that up to 45% of patients with anginal symptoms requiring a diagnostic angiogram do not have obstructive coronary artery disease [4, 5]. Almost half of these patients have a positive acetylcholine provocation test, which illustrates that the underlying mechanism in many symptomatic patients is dynamic in nature. It is important to recognise this syndrome as these patients are at risk of developing unstable coronary syndromes and fatal arrhythmias.

It this issue, several aspects of gender differences in coronary artery disease are highlighted to inform the readers about the latest aspects on this topic of interest. A report discussing a questionnaire among cardiologists illustrates the perceived need for systematic approach in this patient category, although it is worth mentioning that only 10% of the cardiologists approached responded [6]. In one of the articles, Beijk et al. advocate a full diagnostic workup of these patients based upon the recommendations of international working groups [3].

At the moment, the European Society of Cardiology (ESC), the American College of Cardiology (ACC) and the American Heart Association (AHA) have no official guidelines for non-obstructive coronary artery disease (NOCAD). The current recommendation for diagnosis and treatment is based upon expert opinions formulated by the international Coronary Vasomotion Disorders International Study Group (COVADIS) [7]. It is conceivable that this proposal will lead to official ESC/ACC/AHA guidelines. This limitation of guideline recommendation has been recognised by the Netherlands Society of Cardiology (NVVC) and resulted in a grant for the Dutch working group Gender to construct a protocol on coronary microvascular dysfunction. It is expected that this protocol will be introduced next year.

One may wonder why it takes so long to make progress in this field of interest in the Netherlands. There are only a few specialised centres in our country. Until recently, the Amsterdam UMC, location Meibergdreef, was the only centre where acetylcholine provocation testing was performed on a routine base. As outlined above, NOCAD is a frequent finding in symptomatic patients undergoing diagnostic coronary angiography, indicating that many patients are misdiagnosed, leading to unnecessary visits to other medical specialist for additional diagnostic tests. This concern about the diagnostic workup, in particular in women with NOCAD, has been highlighted on social media on many occasions. Unfortunately, this has merely fuelled an ineffective debate on the interpretation of symptoms and diagnostic workup in patients with NOCAD.

Hopefully, this issue will contribute to our knowledge of gender differences in coronary artery disease, that includes a diagnostic workup protocol implemented in a few Dutch intervention centres. It is anticipated that this development, together with the progress of national and international working groups, will lead to a rational approach in patients with NOCAD, which is urgently needed for the diagnosis and treatment of this coronary syndrome.

## References

[1] Johnston N, Schenck-Gustafsson K, Lagerqvist B (2011). Are we using cardiovascular medications and coronary angiography appropriately in men and women with chest pain?. Eur Heart J.

[2] Janssen E, de Leeuw PW, Maas A (2019). Spontaneous coronary artery dissections and associated predisposing factors: a narrative review. Neth Heart J.

[3] Beijk MA, Vlastra WV, Delewi R (2019). Myocardial infarction with non-obstructive coronary arteries: a focus on vasospastic angina. Neth Heart J.

[4] Ong P, Athanasiadis A, Borgulya G (2012). High prevalence of a pathological response to acetylcholine testing in patients with stable angina pectoris and unobstructed coronary arteries: The ACOVA Study (Abnormal COronary VAsomotion in patients with stable angina and unobstructed coronary arteries). J Am Coll Cardiol.

[5] Ong P, Athanasiadis A, Hill S (2008). Coronary artery spasm as a frequent cause of acute coronary syndrome: the CASPAR (coronary artery spasm in patients with acute coronary syndrome) study. J Am Coll Cardiol.

[6] Arabis E (2019). Questionnaire survey on cardiologists’ view and management of coronary microvascular disease in clinical practice. Neth Heart J.

[7] Ong P, Camici PG, Beltrame JF (2018). International standardization of diagnostic criteria for microvascular angina. Int J Cardiol.

